# Minimally Invasive Gynecologic Surgery and Enhanced Recovery and Outcomes: A Literature Review

**DOI:** 10.7759/cureus.84814

**Published:** 2025-05-26

**Authors:** Estephano Fabian Ortiz Vazquez, Juan Camilo Londoño Victoria, Gabriela Alejandra Castillo López, Esthela Carolina Hidalgo Tapia, Francisco Isaac Mena Acosta, Sisa Del Rocio Puchaicela Namcela

**Affiliations:** 1 Obstetrics and Gynecology, Hospital Materno Infantil de Tijuana, Tijuana, MEX; 2 Obstetrics and Gynecology, Universidad de Antioquia, Medellín, COL; 3 Outpatient Care, Integramed, Quito, ECU; 4 Health Education, Universidad de Cuenca, Cuenca, ECU; 5 Obstetrics and Gynecology, Universidad del Azuay, Cuenca, ECU; 6 Medical Research, Escuela Superior Politécnica de Chimborazo, Loja, ECU

**Keywords:** clinical outcomes, enhanced recovery after surgery, enhanced recovery after surgery (eras), future directions, minimally invasive gynecologic surgery

## Abstract

Enhanced recovery after surgery (ERAS) protocols have significantly improved clinical outcomes in patients undergoing minimally invasive gynecological procedures. However, the absence of protocol uniformity among institutions, uneven implementation, uneven compliance, and the absence of research randomization limit the generalizability of findings. The aim of the review is to evaluate technological advancement, the efficacy of the integration of ERAS in minimally invasive gynecological surgery (MIGS) to improve clinical outcomes, challenges and controversies, and the way forward.

The implementation of ERAS within minimally invasive gynecologic procedures leads to considerable positive results in postoperative outcomes. The implementation of ERAS protocols produces reduced pain and lower opioid requirements along with earlier patient mobilization and decreased hospital stays and an enhanced potential for same-day discharge for various benign and malignant gynecological procedures performed through laparoscopic, robotic, or vaginal techniques. The effectiveness of ERAS pathways demonstrates consistency among different surgical patient groups, such as adolescent and elderly women and gynecologic oncology patients, thus proving its broad clinical efficiency for current surgical procedures. A major benefit of this review stems from multiple research methods and extensive sample sizes, and multiple clinical settings to improve the universal application of research findings.

The evidence showed that ERAS implementation was cost-effective, which proves it provides clinical and economic benefits. The implementation of ERAS protocols faces continued challenges because of diverse elements such as inconsistent patient adherence, possible publication bias, and insufficient long-term measurements. The studies use differing standardized outcome measures, which creates barriers to making direct outcome comparisons between them. The existing body of evidence supports establishing ERAS as a standard practice because it contributes to improved recovery and enhances both patient experience and healthcare operational effectiveness during minimally invasive gynecologic procedures.

## Introduction and background

In the US, 600,000 benign hysterectomies are performed annually. To cut costs and complications, the American College of Obstetricians and Gynecologists advises minimally invasive gynecologic surgery (MIGS) [1,2]. In enhanced recovery after surgery (ERAS), peri-surgical care, bowel preparation, stoppage of oral intake after midnight, narcotics, analgesia, and delayed transition to oral intake with prolonged bed rest are recommended. This method may have a slow recovery. It is therefore attempted to design quick surgical protocols to preserve normal physiology during surgery, prevent postoperative problems, and enhance patient outcomes without re-hospitalization [2].

The ERAS standardizes pre- and post-surgical treatment for colon surgery patients and reduces metabolic stress response to speed recovery. Internationally, the ERAS program has successfully integrated surgeons, anesthetists, nurses, and assistant healthcare workers into treatment [3]. Studies show that ERAS protocols can be used in many surgical procedures [4].

A recent meta-analysis found that ERAS in gynecologic oncology reduced hospital length of stay (LOS) by 1.6 days, complications by 32%, readmission by 20%, 30-day postoperative mortality by no change, and mean cost savings of $2129 USD per patient [5]. Higher compliance with ERAS gynecologic oncology recommendations reduced LOS and complications in a dose-response relationship [5]. Based on this research, ERAS should be the standard of treatment for gynecological surgical procedures. However, few recent studies have examined global ERAS uptake in gynecologic oncology. The results show that ERAS recommendations are poorly followed, and implementation hurdles persist despite the greatest efforts [5].

Multidisciplinary studies have proved the benefits of the ERAS method. The absence of protocol uniformity among institutions, uneven implementation, and the absence of research randomization limit the generalizability of findings [6]. The MIGS suitability for ERAS protocol implementation suggests that there is a need for a close look at further evaluation of implementation challenges.

The scope of the review is to observe the technological advancement with regard to artificial intelligence (AI) and machine learning models (MLM). It looks at how these advancements in technology improve surgical precision through high visual imaging techniques preoperatively, perioperatively, and postoperatively. The review analyzes particular gynecologic surgical procedures that benefit from MIGS and ERAS, including hysterectomy, myomectomy, endometriosis surgery, ovarian cystectomy, and pelvic organ prolapse surgery. The review investigates both the preoperative optimization and intraoperative strategies and postoperative care components of ERAS protocols. The review evaluates patient-focused results and life quality metrics, analyzes implementation challenges and controversies of MIGS and ERAS, and establishes future research possibilities in this field.

## Review

Technological innovations in MIGS

Artificial Intelligence and Machine Learning

Artificial intelligence, together with machine learning systems, has started evolving the techniques used for preoperative strategies as well as intraoperative choices. Researchers developed a nomogram predictive model in 2021 using multivariable logistic regression analysis based on ERAS protocol compliance that showed remarkable capability to forecast postoperative outcomes and complications (area under the curve (AUC) = 0.906, sensitivity = 0.948), according to Shen et al. [7].

Advanced Imaging Techniques

The development of MIGS received a boost from state-of-the-art imaging tools. Although 3D imaging and augmented reality are not described specifically in the studies we analyzed, robotic devices and laparoscopic technology signify high-resolution and intraoperative imaging integration for better visualization. Research by Yoong et al. [8] and Peng et al. [9] presents enhanced surgical precision and decreased complications as essential benefits from improved imaging modalities in surgical performance.

Robotics and Automation

Robot-assisted surgery remains the most transformative development to appear in MIGS. Bahadur et al. [10], established that robotic hysterectomy performed with ERAS protocols produced enhanced patient outcomes. The ERAS group showed significantly lower docking time (4.82 ± 0.73 vs. 5.31 ± 0.92 minutes), faster tolerance of diet (0.14 ± 0.35 vs. 1.14 ± 0.35 days), and earlier resumption of ambulation (0.42 ± 0.5 vs. 1.26 ± 0.44 days). Time for “fit for discharge” (1.43 ± 0.61 vs 2.97 ± 1.1 days) and LOS (2.85 ± 1.09 vs 3.78 ± 1.29 days) were significantly lower in the ERAS group. Postoperative complications and readmission rates were comparable. Quality-of-life scores favored the ERAS group at postoperative days one and 30. Robotic systems lead to these enhancements due to their mechanical accuracy, together with their workflow optimization capabilities. Research by Relph et al. [11], combined with Smith et al. [12], demonstrates that robotic automation delivers financial efficiency together with decreased hospital resource consumption in gynecological procedures.

Specific gynecologic surgeries benefiting from MIGS and ERAS

The ERAS protocols in minimally invasive gynecologic oncology surgery deliver essential advantages. This includes decreased hospital LOS with higher same-day discharge rates and reduced costs, together with minimized opioid consumption. Both patients and healthcare systems can benefit from the strong performance of ERAS protocols in oncologic minimally invasive surgery environments [13,14].

Robotic-Assisted Hysterectomy

Conditions treated by robotic-assisted hysterectomy are uterine fibroids and abnormal uterine bleeding (benign indications). Research shows that laparoscopic surgeries under ERAS protocols lead to improved recovery results for patients who have gynecologic cancers, which include endometrial, cervical, and ovarian cancers. Peng et al. [9], recorded that patients achieved faster bowel function recovery, together with lower visual analog scale (VAS) pain scores and reduced nausea and vomiting and shorter hospitalization periods, and diminished systemic inflammatory responses. Lambaudie et al. [15], demonstrated that the duration of hospital stay reduces up to 2.5 to 3 days, while the readmission rate is not affected. The ERAS functions as a safe and multidisciplinary framework that strengthens MIGS outcomes. Beyond MIGS benefits, it adds value to outcomes for both benign and malignant gynecological procedures [16].

Total Laparoscopic Hysterectomy (TLH)

Conditions treated by total laparoscopic hysterectomy (TLH) are uterine fibroids, adenomyosis, and abnormal uterine bleeding. The research conducted by Peng et al. [9], revealed that patients involved in ERAS programs obtained accelerated bowel recovery combined with reduced VAS pain and fewer nausea and vomiting symptoms. Lambaudie et al. [15], surgical protocols provide medical evidence for oncology center ERAS programs. The ERAS protocol was associated with decreases in median LOS (2.5 (0 to 11]) days vs. 3 (1 to 14) days, p = 0.002) and proportion of discharged patients at target LOS (45% vs. 24%; p = 0.002). Morbidities occurred in 25% and 26% of the groups with and without ERAS, and readmission rates were 6% and 8%, respectively, without any significant difference. Moreover, the clinical efficiency of ERAS was verified by low rates of postoperative complications and hospital readmission rates among the enhanced recovery after surgery group due to a statistically significant difference in outcomes, as p < 0.05 [17].

Laparoscopic Hysterectomy or Robotic-Assisted With Pelvic/Para-Aortic Lymphadenectomy

Endometrial cancer, cervical cancer, and ovarian tumors are conditions treated by laparoscopic hysterectomy and robotic-assisted pelvic or para-aortic lymphadenectomy. The study by Peng et al. [9] found that ERAS patients acquired faster bowel recovery and diminished VAS pain scores and fewer nausea and vomiting incidents, and required shorter hospital stays, and reduced systemic inflammation. The implementation of ERAS protocols at expert oncology facilities allowed patients to shorten their hospital stay without experiencing additional readmissions or complications [15].

Older adults: Research evidence shows that ERAS protocols help elderly patients experience positive outcomes during gynecologic oncological surgical treatments. Hospital stay durations across patient age groups matched after laparoscopic or robotic-assisted hysterectomy with lymphadenectomy procedures at age 70 and above, according to Nonneville et al. [18]. In advanced-age patients undergoing gynecologic oncological surgeries, science confirms that ERAS treatments deliver effective protected outcomes.

Pediatric Patients: The ERAS approach produces superior results when used for laparoscopic benign gynecologic procedures in pediatric and adolescent patients who are younger than 25 years old. The research by Smith et al. [12] revealed that 95% of patients were eligible for day release following their surgical procedure without any need for readmission and with medication requirements limited to non-opioid drugs, which promoted both security and operational efficiency.

When ERAS protocols apply non-opioid multimodal analgesia for benign laparoscopic gynecologic procedures, they become remarkably effective. Geng et al. [19] observed improvements in pain (quality of recovery (QoR)-40 domain, p = 0.033), lower morphine usage (p = 0.0006), faster ambulation and bowel recovery (p < 0.01), and overall patient satisfaction (p = 0.000). The MIGS recovery pathways demonstrate an increasing implementation of opioid reduction protocols due to this evidence. The advantages of ERAS protocols seem to vary between different patient groups, especially patients who have chronic pelvic pain or opioid dependence, or psychiatric conditions. Research shows that adaptable ERAS protocols combined with risk-tailored modifications need to be developed to improve surgical results in various MIGS patient populations [20].

Vaginal Hysterectomy

Conditions treated by vaginal hysterectomy are uterine prolapse and benign uterine pathology. Research shows that implementing ERAS methods in vaginal hysterectomy procedures for treating uterine prolapse along with benign uterine pathologies results in quantifiable improvements. The hospital stay duration decreased to 22.0 hours from 45.5 hours, resulting in a 51.6% hospital stay reduction, according to Yoong et al. [8], while patient early discharge increased fivefold from 15.6% to 78.0%. The study by Relph et al. [11] detected less urinary catheter use and better discharge planning processes. Erkan et al. [21] found that minor post-treatment symptoms failed to raise readmission rates, which showed that ERAS worked well for both patient safety and cost reduction.

Laparoscopic Myomectomy

Conditions treated by laparoscopic myomectomy are endometriosis, ovarian cysts, tubal pathology, and leiomyomas using the following procedures such as laparoscopic myomectomy, salpingo-oophorectomy, and cystectomy. The implementation of ERAS proves beneficial for all patients who undergo laparoscopic uterine-sparing surgical procedures due to endometriosis and ovarian cysts, and tubal pathologies. The findings of Peterst et al. [22] demonstrated that patients discharged on the same day increased by 9.4% (p = 0.001), while narcotic use decreased by 64% (p < .001), nausea and vomiting declined, and post anesthesia care unit (PACU) stay time shortened by 19 minutes (p = .036), which reflected enhanced patient experiences and faster healing.

ERAS protocols in MIGS

Preoperative Optimization

Patients undergoing ERAS preoperative optimization benefit from physical and psychological practices before their surgical procedure. Research has shown preoperative counseling to be essential for educating patients, reducing their anxiety levels, and controlling their expectations. The research protocol of Bahadur et al. [10] incorporated preoperative counseling with carbohydrate loading for patients, as it reduces the chances of insulin resistance, anxiety, and chances of hyperglycemia postoperatively. Early preparation protocols possess strong relationships with patient compliance rates as documented by Shen et al. [7]. Patient data show that controlled education delivered by the ERAS “gynecology school” program described by Relph et al. [11] reduced hospital stays and improved discharge preparedness.

The implementation of ERAS protocols in gynecology practice enables patients to experience less surgical stress while recovering more swiftly after their procedures. Preoperative counseling combined with opioid-sparing pain control strategies and minimally invasive surgical techniques, as well as early mobilization and feeding, are key success factors. Outcomes from implementing ERAS require interdisciplinary collaboration and active patient involvement in the process, according to research [22,23]. A nationwide Canadian survey found that a total of 41.9% of respondents work in a center with an ERAS program. Adherence to ERAS recommendations was high when it came to engaging patients in the operative processes, changing equipment after a contaminated procedure, discontinuing urinary catheters, and initiating early postoperative mobilization. The ERAS protocol enhanced adherence to preoperative carbohydrate loading, intraoperative fluid management, normothermia, and bowel-regimen adjuncts (p < 0.05). Despite ERAS programming, adherence to some recommendations, such as preoperative fasting and comorbidity optimization, remained low. Most respondents felt that ERAS is safe (98%) and improves outcomes (82%) [24].

Intraoperative Strategies

The ERAS protocols work during surgery to reduce patient bodily stress while achieving optimized surgical conditions. The standardization of surgical procedures included opioid-free pain management systems coupled with the limitation of invasive medical equipment use. According to Geng et al. [19], healthcare providers should maintain non-opioid multimodal analgesic care throughout all periods of perioperative treatment. Lambaudie et al. [15], applied fluid control methods and temperature stabilization practices together with blood loss reduction methods that brought about diminished inflammatory response and accelerated postoperative healing. The promotion of minimal urinary catheter and vaginal packing by Erkan et al. [21], and Yoong et al. [8], aimed to improve patient recovery along with pain reduction.

Hospital stay duration decreased substantially along with IV fluid administration and opioid use after introducing an enhanced recovery protocol (ERP) in pelvic reconstructive surgery (p-values: 0.04, < 0.01, and < 0.0001, respectively) from 29.9 to 27.9 hours, while IV fluid administration went from 2.7 to 1.5 liters. Increased mobility among ERP patients resulted in faster discharge rates without recorded adverse effects among patients. Research evidence showed that patient satisfaction significantly increased without any increase in 30-day hospital costs after implementing ERP [25,26]

Postoperative Care

The main focuses of ERAS postoperative care include early patient movement, swift food introduction through oral routes, along with vomiting prevention and the use of non-opioid analgesia and drain and catheter extraction as soon as possible. According to Bahadur et al. [10], patients who were ambulated early and received early diets spent less time in the hospital. The researchers from Shen et al. [7], along with Geng et al. [19], discovered that patient satisfaction, along with reduced complications, aligned directly with early mobilization combined with oral nutrition. The research by Smith et al. [12] proved that pediatric patients could safely benefit from quick hospital discharge, together with non-narcotic medications. Peterst et al. [22] also observed reduced PACU stays and opioid usage with early feeding and multimodal pain management.

Patient-Centered Outcomes and Quality of Life

Studies indicate that combining ERAS protocols with MIGS generates better outcomes regarding patient-centered results that include shortened recovery time, superior pain management, decreased emotional distress, and greater overall satisfaction ratings. The combination of clinical achievements translates into better quality of life experiences following surgical procedures for patients. Bahadur et al. [10] demonstrated that patients undergoing robotic-assisted hysterectomy under ERAS returned to diet, ambulation, and discharge readiness earlier compared to traditional standards. The outcomes of shorter hospital stays, as well as higher postoperative satisfaction, combined with better comfort, successively impacted patient well-being. Patients managed through ERAS recovery experienced better quality of life results than patients who followed standard postoperative protocols.

Research by Geng et al. [19] examined multimodal analgesic components within ERAS to determine their impact on recovery quality. The researchers discovered statistical improvements in the pain evaluation segment of QoR-40 scores (p = 0.033) alongside reduced opioid drug consumption. Patients who went through ERAS recovered bowel function more quickly and achieved better satisfaction scores while experiencing an easier recovery journey. According to Smith et al., children and young adults experienced 95% same-day discharge and zero readmissions and complications due to the implementation of ERAS protocols [12]. The pain control methods for 95% of patients exclusively relied on non-narcotic medications, which allowed them to remain neutral from side effects, thus creating positive health benefits for their bodies and minds.

Patient satisfaction, together with QoR-15 domains, received significant improvement when ERAS protocols achieved higher compliance, according to Shen et al. [7] (p < 0.001 and p < 0.05). The integration of perioperative care structure and patient training alongside early patient movement resulted in superior comfort, together with emotional well-being after surgical procedures. Total patient costs declined from $8381 to $7252 per patient, while postoperative stay expenses and operating room costs, as well as pharmacy costs, experienced substantial savings (Table 1) [27].

**Table 1:** Primary studies reviewed ERAS: Enhanced recovery after surgery, PACU: Post-anesthesia care unit, LOS: Length of stay

Author and year	Study design	Gynecological condition treated	Groups	Minimally invasive procedure	ERAS protocol	Outcome of procedure
Bahadur et al., 2024 [10]	Randomized controlled trial	Benign indications requiring robotic hysterectomy	ERAS group (n = 65); Conventional (non-ERAS) group (n = 65)	Robotic hysterectomy	The ERAS protocol in the study included: Preoperative counseling, carbohydrate loading, early removal of the catheter, and early ambulation	After robotic hysterectomy, both groups had improved outcomes. The ERAS group had considerably reduced docking time (4.82 ± 0.73 vs. 5.31 ± 0.92 minutes). The ERAS group showed faster diet tolerance (0.14 ± 0.35 vs. 1.14 ± 0.35 days). The ERAS group resumed walking sooner (0.42 ± 0.5 days vs. 1.26 ± 0.44 days). Early "fit for discharge" was observed in the ERAS group (1.43 ± 0.61 vs. 2.97 ± 1.1 days). The ERAS group had a shorter hospital stay (2.85 ± 1.09 days vs. 3.78 ± 1.29 days). The ERAS group lived better.
Shen et al., 2021 [7]	Prospective observational study	Benign conditions requiring hysterectomy	Group I: < 60% compliance (148 patients); Group II: 60–79% compliance (160 patients); Group III: ≥ 80% compliance (167 patients)	Laparoscopic hysterectomy	A modified ERAS protocol included: Postoperative nausea and vomiting (PONV) prophylaxis, early mobilization, early oral nutrition, and early removal of urinary drainage post minimally invasive surgery	Multisystem inflammatory syndrome (MIS) was associated with fewer complications. Higher ERAS compliance (≥80%) is statistically significantly associated with reduced postoperative complications (p < 0.001), shorter hospital stay (p < 0.001), higher patient satisfaction (p < 0.001), and improved QoR-15 domains (p < 0.05). A nomogram model was built with strong predictive power: AUC = 0.906, sensitivity = 0.948
Geng et al., 2021 [19]	Randomized controlled trial	Benign conditions requiring laparoscopic gynecological surgery	Study group: Received multimodal analgesia-based ERAS; Control group: Received conventional opioid-based analgesia	Laparoscopic gynecological surgery	ERAS protocol included: Non-opioid medications across all perioperative phases and early ambulation, rapid return of bowel function	Higher ERAS compliance (≥80%) is significantly linked to lower postoperative complications (p < 0.001), shorter hospital stay (p < 0.001), higher patient satisfaction (p < 0.001), and improved QoR-15 domains (p < 0.05). The nomogram model proved highly predictive: AUC = 0.906, sensitivity = 0.948
Yoong et al., 2014 [8]	Cohort control study	Benign conditions requiring vaginal hysterectomy	Intervention: 50 patients who underwent VH after ERAS implementation; Control group: 50 patients who underwent VH before ERAS implementation	Vaginal hysterectomy	Standardized anesthetic and postoperative management protocols, strategies to minimize the use of invasive devices (e.g., urinary catheters, vaginal packing), systems promoting early recovery (e.g., early mobilization and discharge planning)	Length of stay: 51.6% shorter (22.0 vs. 45.5 hours; p < 0.01). Discharge within 24 hours increased five-fold (78.0% vs. 15.6%; p < 0.05). Urinary catheter and vaginal packing usage decreased and were removed early (p < 0.05 in all cases). No significant changes in readmissions or ED attendance. Saved 9.25% per patient on perioperative costs.
Relph et al., 2014 [11]	Comparative study	Benign conditions treated with vaginal hysterectomy	Intervention: 45 patients undergoing VH after ERAS protocol implementation; Control group: 45 matched patients undergoing VH before ERAS implementation	Vaginal hysterectomy	Specific components: Reduced catheter use, shortened hospital stay, patient education via a gynecology 'school', care coordination by a specialist enhanced recovery nurse	Significant reductions in length of hospital stay (p < 0.05) and catheter use (p < 0.05). Accident and emergency (A&E) attendance increased post-discharge in the ERAS group (minor symptoms) (p < 0.05). No significant increase in inpatient readmission (p > 0.05). Cost savings of £164.86 per patient (15.2%), despite upfront investment in staff and education.
Erkan et al., 2024 [21]	Randomized controlled trial	Benign gynecological diseases treated with laparoscopic hysterectomy	Control group: 50 patients receiving standard care; Study group: 50 patients receiving the ERAS protocol	Laparoscopic hysterectomy	Standard for ERAS: Early mobilization, limited fasting, multimodal analgesia	No significant difference in operative time, bleeding volume, hemoglobin levels, use of drains, and complications. Significant improvements in pain control (VAS scores lower at all time points), postoperative comfort (less nausea and vomiting), hospital stay (shorter), gas passage (earlier), and analgesic demand (reduced).
Lambaudie et al., 2017 [15]	Observational study	Gynecological cancers (endometrial, cervical, ovarian)	Control group: 100 patients receiving standard care; Study group: 100 patients receiving the ERAS protocol	Hysterectomy, pelvic/para-aortic lymphadenectomy	Multimodal perioperative care (nutrition, mobilization, analgesia, etc.)	Decreased median LOS: 2.5 vs. 3 days (p=0.002), higher rate of discharge at target LOS of 2 days: 45% vs. 24% (p=0.002), no significant differences in morbidity (25% vs. 26%) or readmission rates (6% vs. 8%)
Peng et al., 2021 [9]	Randomized controlled trial	cervical tumors, uterine tumors, or ovarian tumors	ERAS group: n = 65; Conventional perioperative care group: n = 65	Laparoscopic procedure	Reduced fasting, early feeding, fluid restriction, lower opioid use, and early mobilization	Faster return of bowel function, significantly less pain (lower VAS scores), less postoperative nausea and vomiting, shorter hospital stay, and lower total cost
Nonneville et al., 2018 [18]	Observational study	Gynecological cancers requiring hysterectomy and/or pelvic/para-aortic lymphadenectomy (likely endometrial, ovarian, or cervical cancers)	Older group: ≥70 years old (n = 75); Younger group: <70 years old (n = 254)	Laparoscopic or robotic-assisted surgery	Shorter fasting, early oral feeding, early mobilization, and opioid-sparing approaches	LOS for patients ≥70 years was similar to younger patients after applying ERAS
Peterst et al., 2020 [22]	Retrospective cohort study	Benign gynecological conditions (tubal/adnexal pathology, endometriosis, leiomyomas)	ERAS group: n = 196; Conventional perioperative care group: n = 214	Laparoscopic uterine-sparing procedures	Early discharge, optimized pain control, antiemetic use, reduced narcotics, and shortened PACU stays	9.4% increase in same-day discharge (p = .001); reduced pain, nausea, and vomiting; 64% reduction in narcotic use (p < .001); 19 min shorter PACU stay (p = .036)
Smith et al., 2020 [12]	Retrospective study	Pediatric and adolescent gynecological conditions	Pediatric and adolescent patients (<25 years old) undergoing laparoscopic or open abdominal (XLAP) gynecologic surgery	85% laparoscopic surgery	Preoperative, intraoperative, and postoperative components, along with follow-up pain assessment	95% discharge on postoperative day 0 (laparoscopic surgery), no complications or readmissions, 95% of patients required only non-narcotic ERAS medications

Challenges and controversies

Variability in ERAS Compliance and Implementation

The main obstacle to implementing ERAS in MIGS originates from inconsistent follow-up of established protocols. Shen et al. [7] grouped patients according to their degree of ERAS compliance, and their research showed improved outcomes when patients met or exceeded 80% adherence. The majority of patients showed poor compliance with ERAS despite having barriers such as unequal protocol implementation, minimal institutional support, patient age, and health conditions.

Managing Postoperative Symptoms Without Opioids

Patient groups may face challenges from the opioid-sparing analgesia aspect of ERAS due to concerns regarding adequate pain management through non-opioid regimens. Geng et al. [19], showed better pain management through multimodal non-opioid analgesic therapy, yet highlighted the need to handle the risk of under-medicating patients, particularly when they present low pain tolerance or past opioid treatment. The issue of attaining similar opioid-free pain management effectiveness lies as a crucial clinical challenge. A complete ERAS protocol for minimally invasive hysterectomy in gynecologic oncology decreased opioid medications while enhancing postoperative pain levels. Clinician adherence to multimodal analgesia techniques alongside complete adherence to ERAS elements proves effective for recovery optimization and lowering opioid dependence. Weston et al. found that the use of multimodal analgesia is an essential part of the ERAS protocol. Multimodal analgesia includes preoperative administration of oral non-opioid analgesics and scheduling acetaminophen, non-steroidal anti-inflammatory drugs (NSAIDs), and gabapentin doses postoperatively to control pain and quicker recovery of patients [28].

Post-Discharge Symptom Management and Readmissions

Although ERAS is associated with early discharge and reduced hospital stay, this has raised concerns about potential increases in post-discharge emergency visits. Relph et al. [11] reported that while overall readmission rates did not increase, minor post-discharge symptoms (e.g., urinary discomfort or mild pain) led to a statistically significant rise in emergency department attendance. This reflects a need for improved post-discharge support and patient education. Researchers implemented same-day discharge as the standard discharge plan with ERAS orders and educational material distribution, which resulted in same-day discharge rates increasing from 14% to 82% (odds ratio, 28; p < .001). The successful improvement in same-day discharge outcomes did not result in postoperative emergency department visits since they remained steady at 8% within seven days, followed by 12% within 30 days [29]. Various studies focused on outpatient minimally invasive hysterectomy for benign conditions showed a 60% rate of same-day discharge and a 3% mean readmission rate among patients from the United States. These studies confirmed that same-day discharge patients experienced neither higher postoperative complications nor hospital readmissions compared to inpatients. The successful implementation of same-day discharge depended on patient age being under 45 years old and expedited surgical timing and shortened operating times, as patients reported high satisfaction levels [30].

Application in Older or High-Risk Populations

The applicability of ERAS in elderly or medically complex patients is sometimes questioned. Nonneville et al. [18] demonstrated that ERAS is feasible in patients ≥70 years old undergoing gynecologic oncology surgery, with outcomes comparable to younger patients. However, the study also implies the need for tailored protocols, as older patients may present with different recovery trajectories, comorbidities, or tolerance levels that challenge standard ERAS timelines.

Institutional and Resource Limitations

Effective ERAS implementation often depends on institutional infrastructure, interdisciplinary coordination, and trained personnel. Relph et al. [11] noted that while ERAS reduced costs long-term, it required upfront investments in staff education and program development (e.g., recovery nurses and patient education programs). For resource-limited settings, these initial costs and workflow restructuring can be a significant barrier to adoption.

Future directions and research opportunities

Enhancing ERAS Compliance and Personalization

Future efforts should focus on improving ERAS protocol compliance through better patient engagement, real-time monitoring, and team-based implementation strategies. Shen et al. [7] showed a strong association between higher compliance (≥80%) and improved outcomes. The association ensures the value of patient-centered care, commitment to quality care, and ethical responsibility to ensure that all patients receive adequate and appropriate care due to the ERAS protocol. However, variability across patient groups suggests the need for personalized ERAS pathways that adapt to individual risk profiles, including age, comorbidities, and surgical complexity.

Expanding the Role of Predictive Analytics and AI

The development of a predictive nomogram by Shen et al. [7] to estimate complication risk and outcomes represents a step toward AI-driven decision support in MIGS. Expanding on this model could lead to more refined tools that predict patient recovery trajectories, flag risks preoperatively, and guide resource allocation, paving the way for precision medicine in surgical recovery planning.

Standardization and Global ERAS Guidelines for MIGS

Although ERAS is effective, differences in protocol elements across studies (e.g., dietary instructions, catheter use, and discharge timing) highlight the need for standardized global guidelines for MIGS. Studies like those by Bahadur et al. [10], Yoong et al. [8], and Relph et al. [11] used institution-specific adaptations. Future research should aim to harmonize ERAS components specific to gynecologic surgery, validated across diverse populations and healthcare systems.

Long-Term Quality of Life and Functional Outcomes

Most studies, including those by Geng et al. [19] and Smith et al. [12], focus on short-term outcomes such as pain control, discharge timing, and early recovery. Future research should explore long-term quality of life, functional recovery (e.g., return to work or sexual function), and emotional well-being to fully understand the impact of ERAS beyond the immediate postoperative window.

Integration of ERAS in Underserved and High-Risk Populations

There is a growing opportunity to evaluate ERAS outcomes in underserved, elderly, and high-risk populations. Nonneville et al. [18] provided evidence for the safe application of ERAS in elderly oncology patients, but further studies should investigate protocol adaptation for frail individuals, those with multimorbidity, or those in resource-limited environments.

Technological Innovations to Support ERAS

With ongoing advancements in surgical technology, incorporating tools such as wearable recovery monitors, telehealth follow-up, and robotic automation could enhance ERAS outcomes. Bahadur et al., 2024 [10], have already demonstrated the efficiency improvements of robotic surgery. Future research could explore automation-enhanced ERAS pathways to further boost recovery speed, monitor patient progress remotely, and reduce the burden on clinical staff.

## Conclusions

The implementation of ERAS within minimally invasive gynecologic procedures leads to considerable positive results in postoperative outcomes. The ERAS protocols ensure reduced pain and lower opioid requirements along with earlier patient mobilization and decreased hospital stays and an enhanced potential for same-day discharge for various benign and malignant gynecological procedures performed through laparoscopic, robotic, or vaginal techniques. The effectiveness of ERAS pathways demonstrates consistency among different surgical patient groups, such as adolescent and elderly women and gynecologic oncology patients, thus proving broad clinical efficiency for current surgical procedures.

A major benefit of this review stems from the multiple research methods, extensive sample sizes, and multiple clinical settings of the studies included. This ensures the universal application of the research findings. The evidence shows that ERAS implementation is cost-effective, which proves it provides clinical benefit and economic value. The implementation of ERAS protocols faces continued challenges because of diverse elements, such as inconsistent patient adherence, possible publication bias, and insufficient long-term measurements. The studies use differing standardized outcome measures, which creates barriers to making direct outcome comparisons between them. The existing body of evidence supports establishing ERAS as a standard practice because it contributes to improved recovery and enhances both patient experience and healthcare operational effectiveness during minimally invasive gynecologic procedures.
